# Supplementary figures and images for: Biochemical, genetic and transcriptional characterization of multibacteriocin production by the anti-pneumococcal dairy strain Streptococcus infantarius LP90
Source: PLoS One. 2020 Mar 5;15(3):e0229417. doi: 10.1371/journal.pone.0229417 (PMC7058333; doi:10.1371/journal.pone.0229417)

## Slide 1
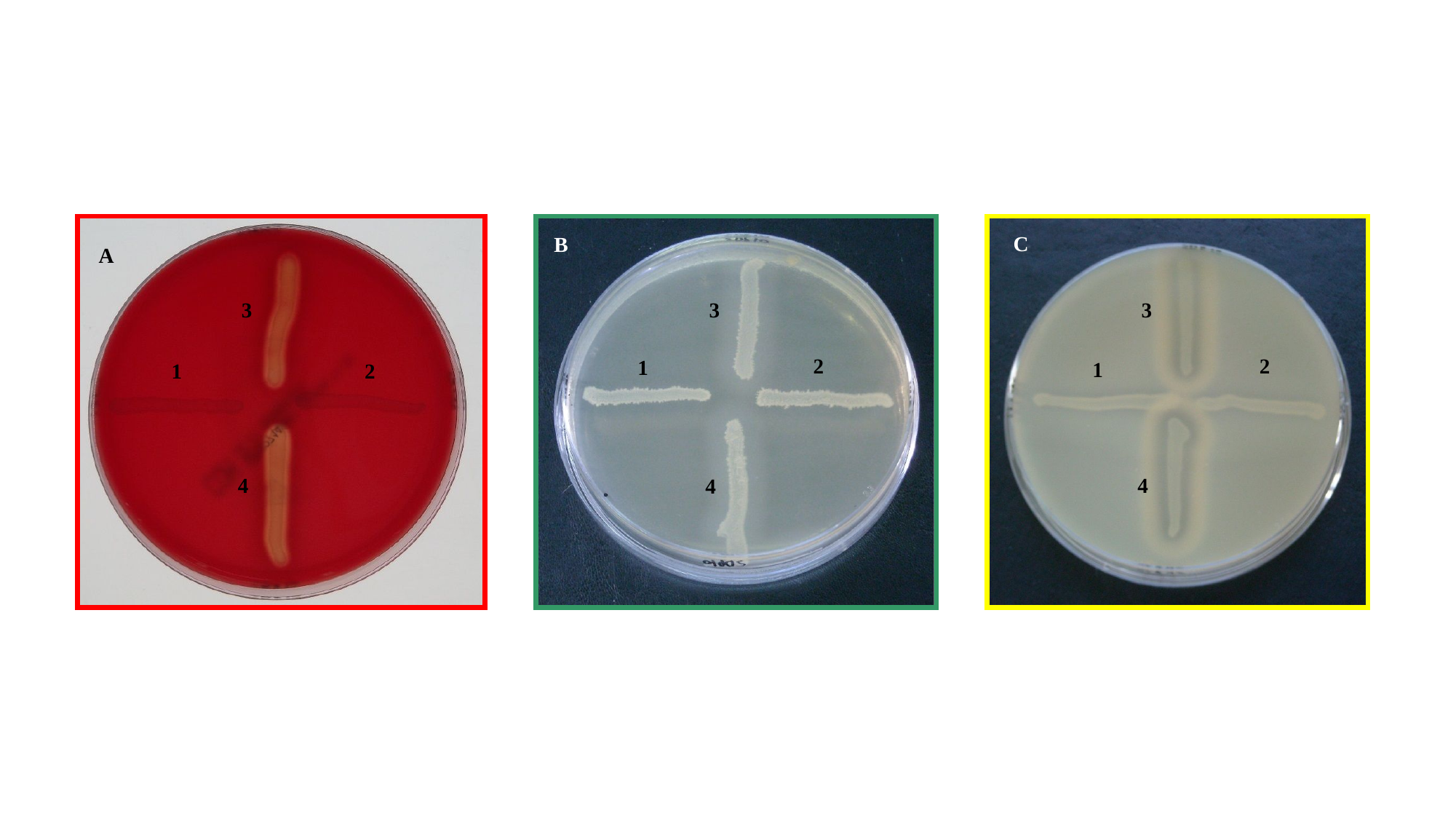

A
B
C
3
3
3
2
2
1
1
1
2
4
4
4

Supplement: S1 Fig — (A) β-hemolysin, (B) gelatinase and (C) protease activity; (1) and (2) S. infantarius LP90, (3) E. faecalis P4, and (4) E. faecalis P36 used as positive controls. (PPT) [file pone.0229417.s001.ppt]

## Slide 1
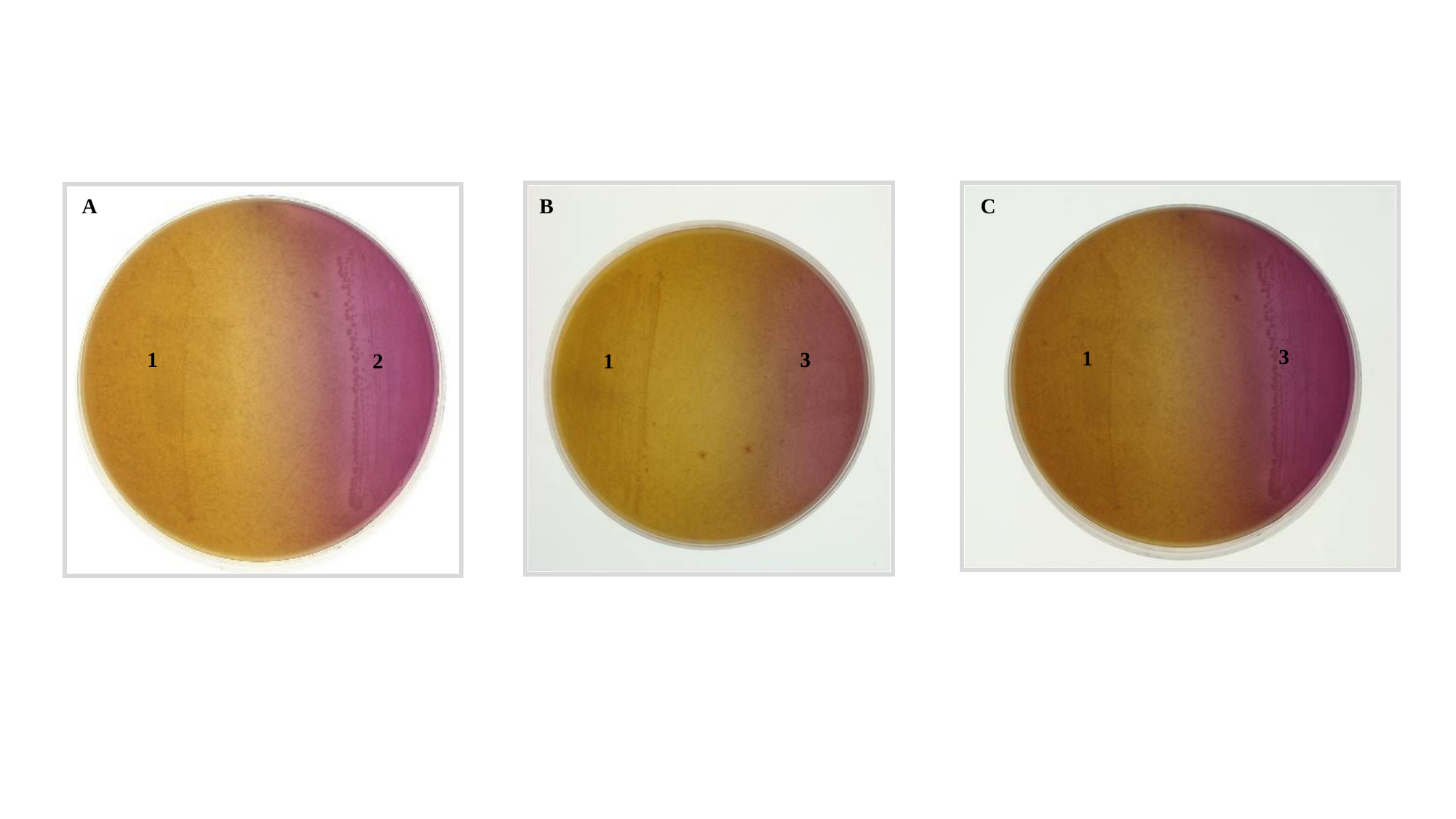

1
2
A
C
B
3
1
3
1

Supplement: S2 Fig — (A) Tyrosine, (B) histidine, and (B) ornithine decarboxylase activities. (1) S. infantarius LP90, (2) Lb. brevis CECT4121 used as positive control for tyrosine descarboxylase activity, and (3) Lactobacillus sp. ATTCC30a used as positive control for histidine and ornithine descarboxylase activities. (PPT) [file pone.0229417.s002.ppt]

## Slide 1
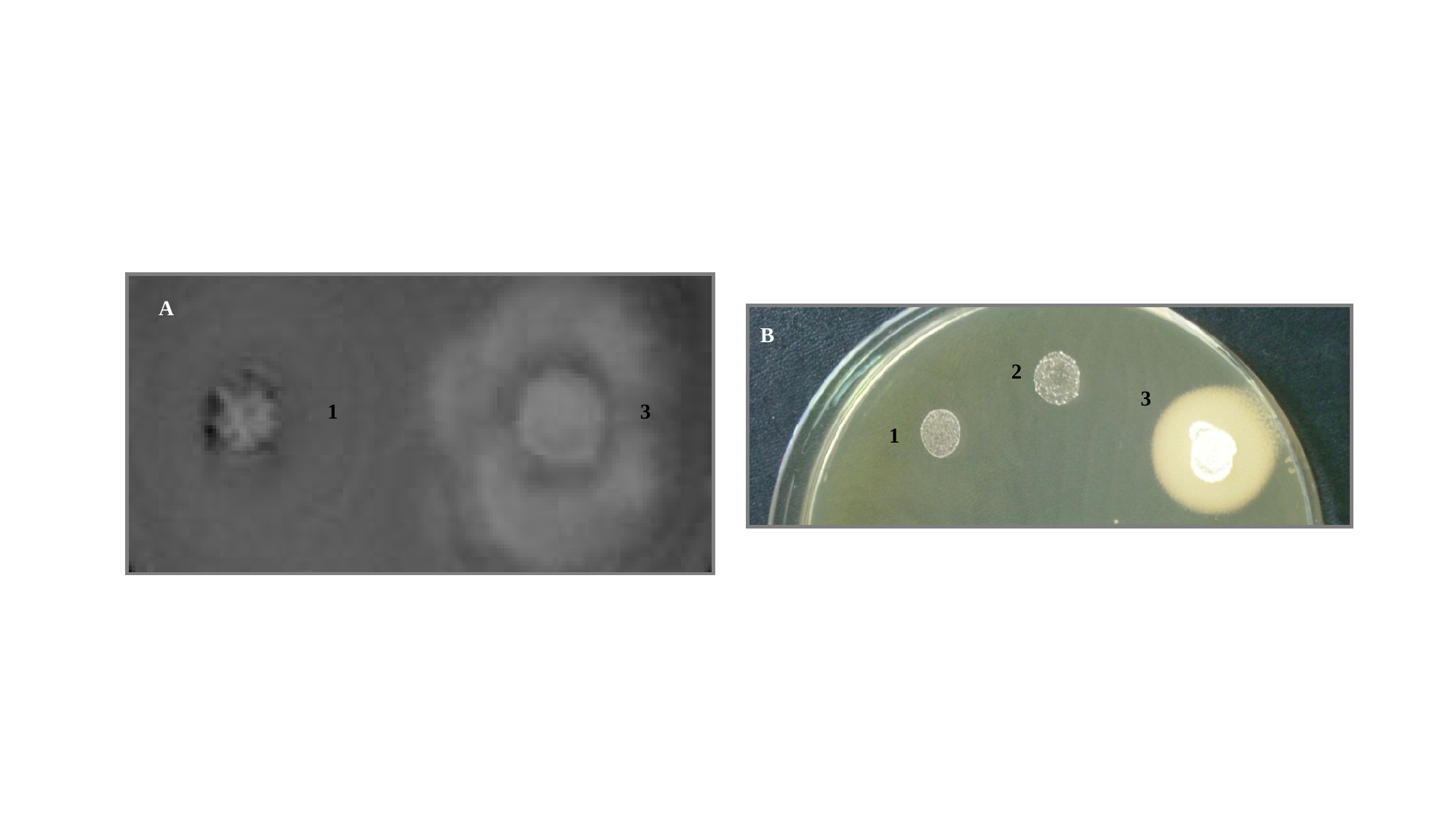

A
B
2
3
1
3
1

Supplement: S3 Fig — Mucine degradation (A) and bile salt deconjugation (B) abilities in S. infantarius LP90. (1) and (2) S. infantarius LP90, and (3) fresh fecal slurry from a healthy adult horse as positive control. (PPT) [file pone.0229417.s003.ppt]

## Slide 1
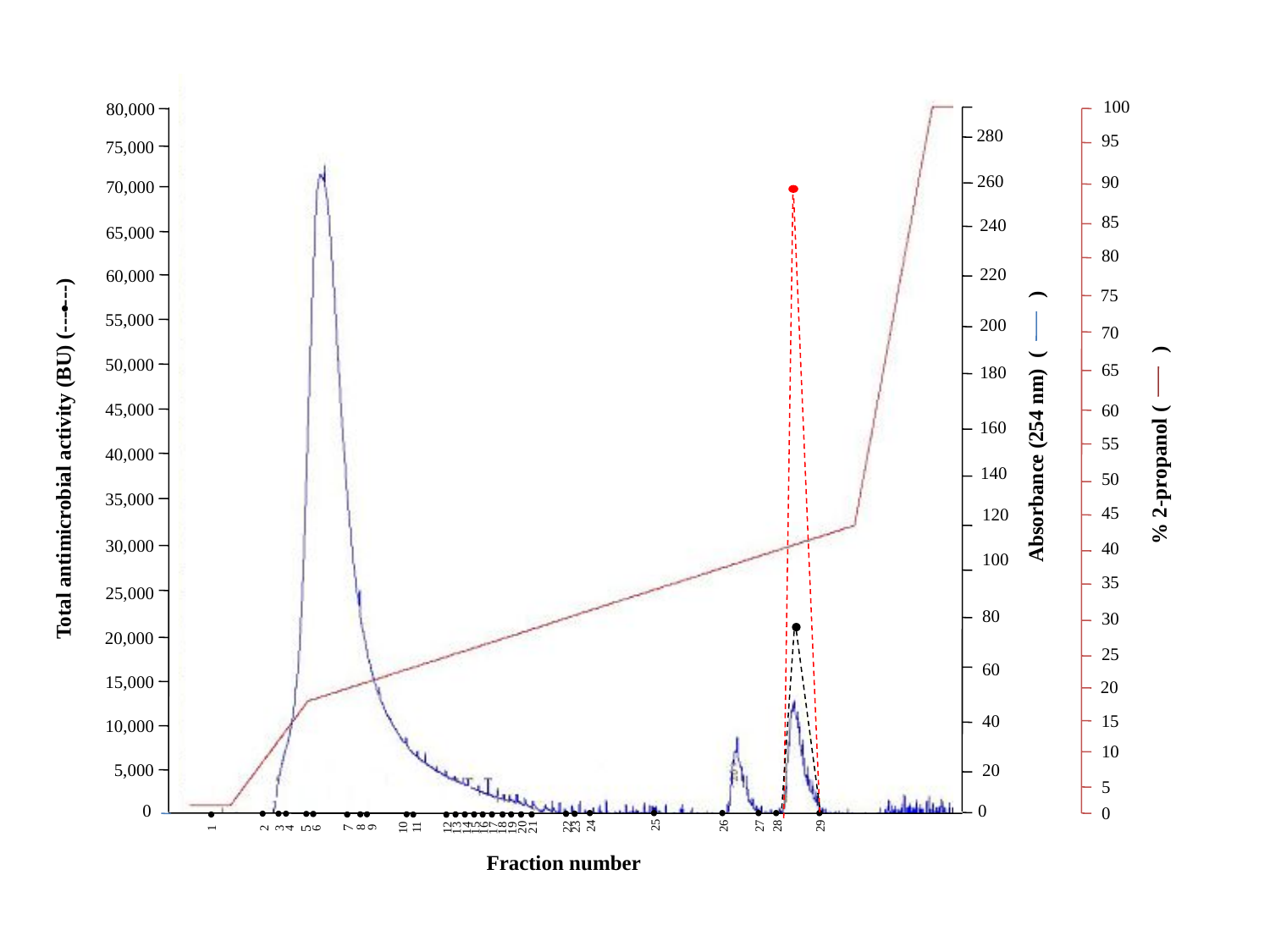

100
80,000
280
95
75,000
260
90
70,000
Absorbance (254 nm) ( )
85
240
65,000
80
220
60,000
Total antimicrobial activity (BU) (--- ---)
75
55,000
200
70
% 2-propanol ( )
50,000
65
180
45,000
60
160
55
40,000
140
50
35,000
45
 120
30,000
40
 100
35
25,000
 80
30
20,000
25
 60
15,000
20
 40
15
10,000
10
 20
 5,000
5
0
0
0
25
24
26
27
28
29
22
23
7
8
11
9
20
21
5
12
10
2
3
4
6
13
14
15
16
17
18
19
1
Fraction number

Supplement: S4 Fig — Fractions 26 and 28 eluted at 30 and 32% (v/v) 2-propanol in aqueous TFA (0.1%, v/v), respectively. Red and black dashed lines indicate antimicrobial activity against S. pneumoniae 66405 and P. damnosus CECT4797, respectively. (PPT) [file pone.0229417.s004.ppt]

## Slide 1
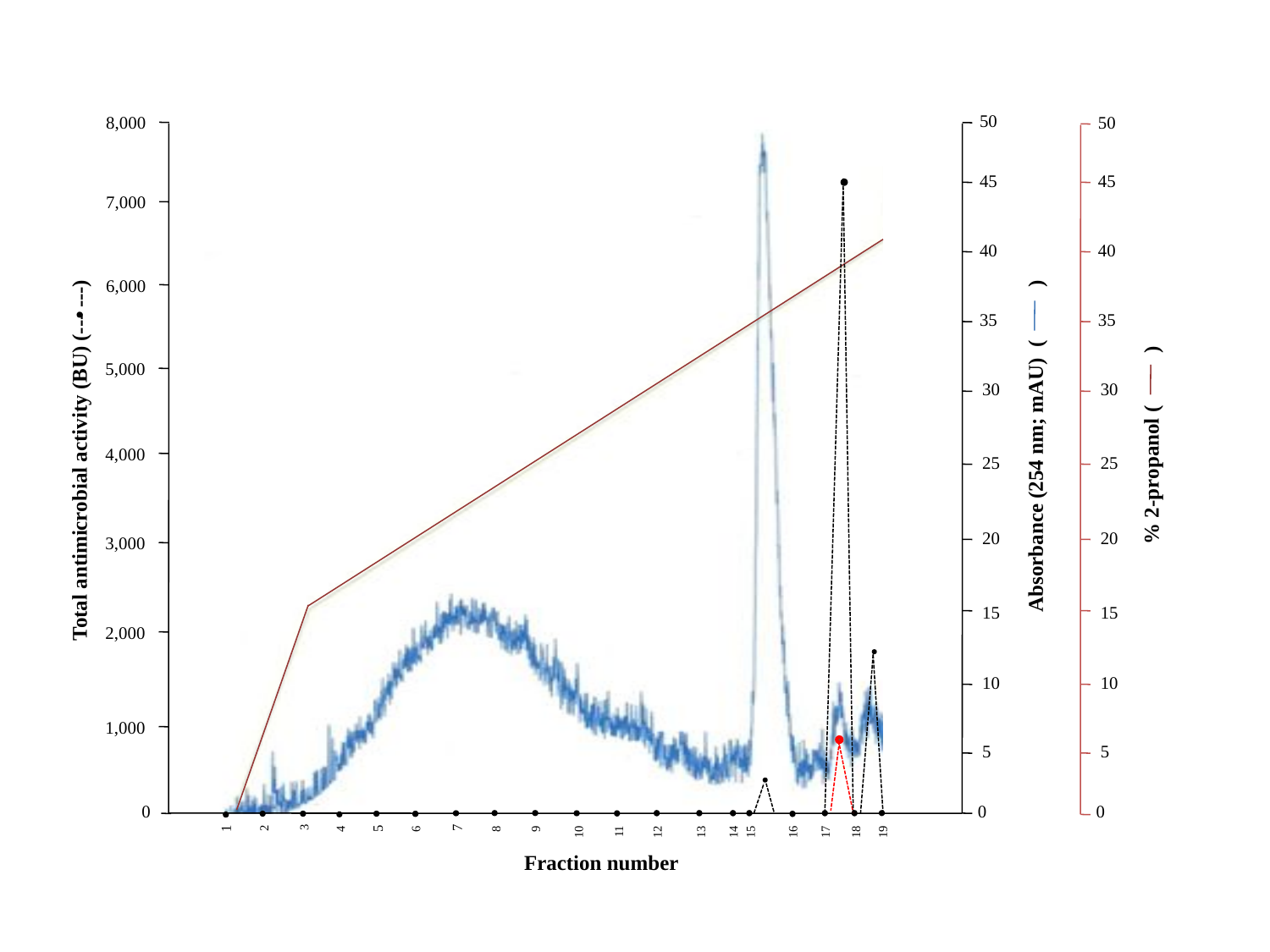

50
50
8,000
45
45
7,000
40
40
Absorbance (254 nm; mAU) ( )
Total antimicrobial activity (BU) (--- ---)
6,000
35
35
% 2-propanol ( )
5,000
 30
 30
4,000
 25
 25
 20
 20
3,000
 15
 15
2,000
 10
 10
1,000
 5
 5
0
0
0
7
1
3
5
2
8
9
4
6
11
10
12
13
14
15
16
17
18
19
Fraction number

Supplement: S5 Fig — Fractions 15, 17, and 18 eluted at 34, 36, and 38% (v/v) 2-propanol in aqueous TFA (0.1%, v/v), respectively. Red and black dashed lines indicate antimicrobial activity against S. pneumoniae 66405 and P. damnosus CECT4797, respectively. (PPT) [file pone.0229417.s005.ppt]
