# Supplementary material for: Biochemical, genetic and transcriptional characterization of multibacteriocin production by the anti-pneumococcal dairy strain Streptococcus infantarius LP90
Source: PLoS One. 2020 Mar 5;15(3):e0229417. doi: 10.1371/journal.pone.0229417 (PMC7058333; doi:10.1371/journal.pone.0229417)
Supplement: Attachment — Submitted filename: PLOS ONE 30374.docx [file pone.0229417.s010.docx]

PLOS ONE: PONE 30374

Biochemical, genetic and transcriptional characterization of multibacteriocin production by the anti-pneumococcal dairy strain *Streptococcus infantarius* LP90

The authors describe the identification of several novel bacteriocins produced by *S. infatarius* LP90, including a bacteriocin with anti-pneumococcal activity. The authors present data to show that the strain LP90 is safe, and fully characterize the gene cluster encoding several potential bacteriocins. These results support the potential for using bacteriocins as alternatives or supplements to antibiotic treatment for combating human infections. The authors need to address the specific points listed below.

1. Page 7: Table 1, showing susceptibility to antibiotics, is inserted within the Materials and Methods section, but it should be moved to Page 15 where these results are reported in the Results section. A list of the antibiotics used could be provided within the text of the Materials and Methods.
2. Page 15, line 315: It should be mentioned in the text if the 37 LAB screened were novel isolates from this study or a previously reported study. Why is reported that LAB inhibited most of the 9 *S. pneumoniae* strains screened, when 23 clinical isolates were tested (lines 316-318)? Was there something unique about these 9 strains, and which 9 strains are being referenced? Line 327: How many strains were inhibited by LP90, which showed the broadest anti-pneumococcal spectrum?
3. Line 342: It should be made clear that LP90 was screened for the presence of known bacteriocin genes by PCR, and none were identified. It should also be said if the streptococcal genes screened for included known bacteriocins from other *S. infantarius* strains.
4. Page 17: Nucleotide sequence of multibacteriocinogenic gene cluster: The labeling of genes are not clear in Figure 3, this image must be generated so the reader can see where the genes and operons are located within the gene cluster. It is difficult to follow the text without the corresponding map of the gene cluster.
5. Line 432: Pediococcus must be spelled out for strain CECT4797. This is the first time this strain is referenced in the text, but it should included within the Materials and Methods section as well.
6. Line 439-444: The fractions reported should be listed specifically with the image shown in Figure 5 (5a, 5b, 5c). The differences between each figure should also be explained with a figure legend. Two lower intensity peaks are reported for fractions B and C, but several other molecular weights are shown, why were these two peaks reported?
7. Line 447: Within the text the different fractions shown in Figure 6 (a and b) should be specified.
8. Line 477: Is it correct to say the potential bacteriocins “would share the same secretion and regulation system”? Is this based on the presence of similar signal peptides on bacteriocins, or just on the fact that they are co-located within the same gene cluster? The authors need to be clear when making conclusions within the Discussion. Line 486-487: Which bacteriocins are potentially class IIa and IIe, since three bacteriocins are listed.
9. Line 496-501: What is shown that orf29 encoded an IF, or was this conclusion based on sequence similarity? Are the molecular masses variants of InfA_1_ or could they be separate peptides? This section, describing the peptides with molecular masses of 3, 963; 3,354 and 3.357 Da, needs to be discussed more clearly.
