# Supplementary material for: Biochemical, genetic and transcriptional characterization of multibacteriocin production by the anti-pneumococcal dairy strain Streptococcus infantarius LP90
Source: PLoS One. 2020 Mar 5;15(3):e0229417. doi: 10.1371/journal.pone.0229417 (PMC7058333; doi:10.1371/journal.pone.0229417)
Supplement: Attachment — Submitted filename: PONE-D-19-30374 Response to Reviewers.docx [file pone.0229417.s011.docx]

**PLOS ONE: PONE-D-19-30374**

Biochemical, genetic and transcriptional characterization of multibacteriocin production by the anti-pneumococcal dairy strain *Streptococcus infantarius* LP90

**ACADEMIC EDITOR**

Dear Dr. Anirudh K. Singh,

Thank you very much for your criticism and constructive comments on our manuscript. As you suggested, a higher resolution Figure 5 has been provided and this image was showed to some colleagues that understood and interpreted it without difficulty.

Sincerely yours

Ass. Prof. Luis M. Cintas Izarra

**REVIEWER #1:**

Dear Reviewer:

Thank you very much for your criticism and constructive comments on our manuscript. Please, find below our point-to-point answers to your comments:

1. Line 80: OD600, 600 as subscript.

Modified as suggested (lines 80, 95 and 300; pages 4, 5 and 13, respectively)

2. Lines 308-309: Could the authors explain which reference gene was used for RT PCR? And which method was applicated for the analysis?

In all the RT-PCR analysis we obtained amplicons of the expected size, which was used as an internal control that the reactions were working properly. On the other hand, with regard to the method applied for the analysis, please note that a paragraph with this information has been included in the revised manuscript (lines 304-310, page 14).

3. Lines 331-339 and 342-343: It is necessary to write “not shown” several times? The authors can show positive results.

As suggested, these results have been included in the revised manuscript as supplemental files (S1 Fig, S2 Fig and S3 Fig).

4. Line 440: Could authors show the three peaks obtained?

We are not sure if you want to see only this chromatogram or also the other obtained with the purification procedure 1. Please note that both chromatograms have been included as supplemental files (S4 Fig and S5 Fig).

5. Supporting Information S4 Table*. S. uberis* strains yield Nisin U, Uberolisin and Ubericin A genes and *S. uberis* strains could be used as control strains.

We acknowledge that *S.uberis* strains can be used as bacteriocin positive controls but, unfortunately, we could not have access to these strains.

**REVIEWER #2**

Dear Reviewer:

Thank you very much for your criticism and constructive comments on our manuscript. Please, find below our point-to-point answers to your comments:

1. Page 7: Table 1, showing susceptibility to antibiotics, is inserted within the Materials and Methods section, but it should be moved to Page 15 where these results are reported in the Results section. A list of the antibiotics used could be provided within the text of the Materials and Methods.

A list of the antibiotics used has been introduced in the Materials and Methods section (lines 140-143, page 7).

Table 1 has been deleted from “Material and Methods” and introduced in the Results section (line 343, page 15).

2. Page 15, line 315: It should be mentioned in the text if the 37 LAB screened were novel isolates from this study or a previously reported study.

The sentence “isolated in previously reported works” has been introduced in the Results section (line 318, page 14).The references are in S1 Table.

Why is reported that LAB inhibited most of the 9 *S. pneumoniae* strains screened, when 23 clinical isolates were tested (lines 316-318)?

As stated in the Materials and Methods section, the direct antimicrobial activity of the 37 bacteriocinogenic LAB was screened by a stab-on-agar test (SOAT) against 9 *S. pneumoniae* strains (lines 83-84, page 4), while their extracellular antimicrobial activity was determined by an agar well-diffusion test (ADT) against 23. *S. pneumoniae* strains (lines 92-94, page 5).

Was there something unique about these 9 strains, and which 9 strains are being referenced?

These 9 strains were selected on the basis of their serotypes and antibiotic susceptibility patterns to assure heterogeneity. The information about which 9 strains were used as indicators in SOATs can be found in S2 Table.

Line 327: How many strains were inhibited by LP90, which showed the broadest anti-pneumococcal spectrum?

Please, note that this information has been included in the revised manuscript (lines 331-332; page 15).

Line 342: It should be made clear that LP90 was screened for the presence of known bacteriocin genes by PCR, and none were identified.

Please, note that this subject has been clarified in the revised manuscript (lines 346-347; page 16).

3. It should also be said if the streptococcal genes screened for included known bacteriocins from other *S. infantarius* strains.

Bacteriocins have not been previously identified in the species *S. infantarius*. Please, note that this sentence has been included in S4 Table footnote (superscript b explanation).

4. Page 17: Nucleotide sequence of multibacteriocinogenic gene cluster: The labeling of genes are not clear in Figure 3, this image must be generated so the reader can see where the genes and operons are located within the gene cluster. It is difficult to follow the text without the corresponding map of the gene cluster.

Please note that this figure has been modified to increase its resolution.

5. Line 432: *Pediococcus* must be spelled out for strain CECT4797. This is the first time this strain is referenced in the text, but it should include within the Materials and Methods section as well.

As suggested, *Pediococcus* has been spelled in the Materials and Methods section (line 262, page 12).

6. Line 439-444: The fractions reported should be listed specifically with the image shown in Figure 5 (5a, 5b, 5c). The differences between each figure should also be explained with a figure legend.

Listed as suggested (lines 443-453, page 24). Moreover, the differences between each figure have been explained in Figs 5 legends. Please, note that the predominant peaks are pointed with an arrow in the modified Figs 5.

Two lower intensity peaks are reported for fractions B and C, but several other molecular weights are shown, why were these two peaks reported?

This sentence has been deleted. Because the reported peaks have molecular masses which could be corresponded to modified (oxidized) bacteriocins as indicated in the Discussion section (lines 521-530, page 27 and 28).

7. Line 447: Within the text the different fractions shown in Figure 6 (a and b) should be specified.

The different fractions shown in Figs 6a and b have been specified as suggested (lines 459-460, page 25).

Please, note that the letter “a” has been added to Fig.6 (line 526, page 27; and line 536, page 28).

8. Line 477: Is it correct to say the potential bacteriocins “would share the same secretion and regulation system”? Is this based on the presence of similar signal peptides on bacteriocins, or just on the fact that they are co-located within the same gene cluster? The authors need to be clear when making conclusions within the Discussion.

The bacteriocins are clustered and flanked by secretion and regulation genes, which supports that they would share the same secretion and regulation systems.

Line 486-487: Which bacteriocins are potentially class IIa and IIe, since three bacteriocins are listed.

This has been clarified in the revised manuscript (lines 496-497, page 26).

9. Line 496-501: What is shown that orf29 encoded an IF, or was this conclusion based on sequence similarity?

This statement is based on sequence similarity. Please, note that this has been clarified in the revised manuscript (line 506-507, page 27).

Are the molecular masses variants of InfA_1_ or could they be separate peptides? This section, describing the peptides with molecular masses of 3, 963; 3,354 and 3.357 Da, needs to be discussed more clearly.

We agree that these two peptides could be separate peptides and have mentioned this possibility in the revised manuscript (lines 508-509, page 27). However, we do consider more likely the hypothesis we present in the manuscript. With regard to this, we would like to note that some bacteriocins require a non-peptidic moiety to exert antimicrobial activity. On the other hand we have done our best to modify the section describing the peptides with molecular masses of 3, 963 and 3,354 Da in order to make it more clear for the reader (lines 508-522, page 27).
